# Failure to Rescue in Major Abdominal Surgery: A Regional Australian Experience

**DOI:** 10.1007/s00268-023-07061-x

**Published:** 2023-05-24

**Authors:** Pranav Divakaran, Joshua Sungho Hong, Saleh Abbas, Stella-May Gwini, Sonalmeet Nagra, Douglas Stupart, Glenn Guest, David Watters

**Affiliations:** 1 Department of Surgery, University Hospital Geelong, Barwon Health 272-322 Bellarine Street and Ryrie Street, Geelong, VIC 3220 Australia; 2 Warrnambool Base Hospital, Southwest Healthcare, 25 Ryot Street, Warrnambool, VIC 3280 Australia; 3 Geelong Clinical School, Deakin University School of Medicine, Little Malop Street, Geelong, VIC 3220 Australia; 4 Biostatistics Support Service, Level 2 Kitchner House, University Hospital Geelong, Barwon Health 272-322 Bellarine Street and Ryrie Street, Geelong, VIC 3220 Australia

## Abstract

**Background:**

Failure to rescue (FTR) is increasingly recognised as a measure of the quality care provided by a health service in recognising and responding to patient deterioration. We report the association between a patient’s pre-operative status and FTR following major abdominal surgery.

**Methods:**

A retrospective chart review was conducted on patients who underwent major abdominal surgery and who suffered Clavien–Dindo (CDC) III-V complications at the University Hospital Geelong between 2012 and 2019. For each patient suffering a major complication, pre-operative risk factors including demographics, comorbidities (Charlson Comorbidity Index (CCI)), American Society of Anaesthesiology (ASA) Score and biochemistry were compared for patients who survived and patients who died. Statistical analysis utilised logistic regression with results reported as odds ratios (ORs) and 95% confidence intervals (CIs).

**Results:**

There were 2579 patients who underwent major abdominal surgery, of whom 374 (14.5%) suffered CDC III-V complications. Eighty-eight patients subsequently died from their complication representing a 23.5% FTR and an overall operative mortality of 3.4%. Pre-operative risk factors for FTR included ASA score ≥ 3, CCI ≥ 3 and pre-operative serum albumin of < 35 g/L. Operative risk factors included emergency surgery, cancer surgery, greater than 500 ml intraoperative blood loss and need for ICU admission. Patients who suffered end-organ failure were more likely to die from their complication.

**Conclusion:**

Identification of patients at high risk of FTR should they develop a complication would inform shared decision-making, highlight the need for optimisation prior to surgery, or in some cases, result in surgery not being undertaken.

**Supplementary Information:**

The online version contains supplementary material available at 10.1007/s00268-023-07061-x.

## Introduction

Complications following major surgery are relatively common and are estimated to occur in 18–23% of patients [1]. The management of complications is challenging for both the patient and the perioperative team, and adds considerably to the cost of care [2, 3] particularly when further interventions involve readmission, unplanned admission to an intensive care unit (ICU), interventional radiology and/or an unplanned return to theatre (RTT).

Up to a third of patients undergoing surgery for cancer of the gastrointestinal (GI) tract, pancreatic and hepatobiliary systems suffer complications [4], with about 14% experiencing severe (Clavien–Dindo grade 3–5) complications [5]. Major complications associated with death have been reported following up to five per cent of elective laparotomies and 33% of emergency laparotomies [6]. The Australia and New Zealand Emergency Laparotomy Audit (ANZELA-QI 2021) reported a mortality rate of 7.1% (range 2.3%–13.3%) [7]

Failure to rescue (FTR), which refers to death after a serious, potentially preventable complication following a surgical procedure, was a concept first introduced by Silber et al. in 1992, to describe a hospital’s proficiency in managing complications after surgical procedures. It has since been adopted widely across various medical specialities as an important quality metric, providing a different perspective from the overall mortality rate [8–10].

There are many factors that influence a patient’s ability to survive a complication of major surgery. These include pre-operative nutritional status [11–13], with serum albumin being a measure of nutritional status [14], a marker of hepatic synthetic function [15, 16] and/or inflammation [17]. Hypoalbuminemia has been reported to be an independent predictor of increased morbidity and mortality, particularly following major abdominal surgery [11, 12, 14, 18–24]. The REASON trial of patients undergoing major non-cardiac surgery reported albumin < 30 g/L to be an important marker of risk and associated poor outcomes [25]. Comorbidities, often measured in combination using the Charlson Comorbidity Index (CCI), are also associated with the risk of death following surgery [26, 27]. Other factors that have been previously shown to be associated with FTR include hospital characteristics such as nurse-to-patient ratios [28], teaching or non-teaching hospital [29] and medical technology availability [30, 31].

Identification of patients with the above-mentioned factors that influence FTR would better prepare surgeons to anticipate complications, physiologically optimise at risk patients where possible or not operate on patients deemed to be at high risk of FTR. This study aimed to assess the pre-operative, operative and post-operative risk factors influencing the ability to rescue a patient suffering from complications after major abdominal surgery.

## Materials and methods

We performed a retrospective review of adult patients (aged 16 years and older) undergoing major emergency and elective abdominal surgery, and who suffered severe complications defined as Clavien–Dindo Classification (CDC) 3–5 [32, 33], after major emergency and elective abdominal surgery. Patients were identified through the Barwon Health General surgical audits from 2012 to 2019. We included both open and laparoscopic operations. We defined major abdominal surgery to include laparotomy, small bowel and/or colonic resection (inclusive of stoma formation, with or without anastomosis), reversal or formation of ileostomy or colostomy, oesophagectomy, gastrectomy, sleeve gastrectomy, gastric bypass, all hepatic resections and all pancreatic surgery.

Abdominal wall hernia repairs, laparoscopic cholecystectomies and appendicectomies were excluded from our definition of “major abdominal surgery”. Patients who underwent radiological, endoscopic or surgical interventions as a consequence of a surgical complication were considered to have suffered a CDC grade 3 complication regardless as to whether anaesthesia was required.

Patient demographic and clinical characteristics, 30-day mortality and data on complications were extracted from the patients’ electronic medical record. FTR was defined as death due to post-operative complications within 30 days of the initial operation and/or death in hospital. All patients who died following surgery were individually reviewed by two surgeons to ascertain if the deaths were a true result of a complication of surgery or if the death was the outcome of an unsalvageable pre-existing disease process. Data extracted included age, sex, body mass index (BMI), comorbidities, smoking status, pre-index surgery lymphocyte count, pre-index surgery serum albumin level and surgical resection for cancer, CCI, the American Society of Anesthesiologists (ASA) score [34, 35], ICU admissions and duration of surgery. Duration of surgery was divided into three subgroups: Under fours, four to eight hours and over eight hours. Blood loss volume data were collected and categorised as greater than or less than 500 mls.

Data were analysed using Stata Statistical Package version 16 (StataCorp. 2019. Stata Statistical Software: Release 16. College Station, TX: StataCorp LLC.) Categorical data were summarised using frequencies and percentages, while continuous/interval data were summarised either as means (with standard deviations; SD) or medians (with 25th and 75th percentiles; P25 and P75, respectively) if data were skewed. FTR mortality rate was reported as a percentage with the Clopper–Pearson binomial confidence interval (CI) [36]. The relationship between FTR and patient characteristics was examined using logistic regression, and the results were reported as odds ratios (ORs) with the 95% confidence interval (CI). Cluster sandwich error estimators were used to account for the lack of independence of measures due to multiple abdominal surgeries for some patients. Both unadjusted and adjusted estimates were reported. The adjusted estimated were obtained by accounting for age, gender, BMI, smoking status, cancer and hypertension diagnosis, pre-operative lymphocytes and albumin, ASA scores and Charlson comorbidity index in the regression model.

The project received ethics approval from Barwon Health’s Human Research Ethics Committee.

## Results

There were 2579 patients who underwent a major abdominal procedure. Of these, 374 (15%) suffered CDC 3–5 complications. Eighty-eight of the 374 patients died, so the FTR rate was 25% (95% CI 19.3%–28.2%) with an overall mortality of 3.4% (95% CI 2.8%–4.2%). Sixty-three of the 88 deaths (72%) died within 30 days of the initial surgery; a further 25 patients (28%) died in hospital after a more prolonged stay.

Table 1 summarises the relationship between demographic/pre-operative patient characteristics and FTR. Higher CCI, ASA score of 4 and low pre-operative albumin levels were significantly associated with FTR. None of the other factors showed significant association on multivariant analysis.

**Table 1:** Relationship between failure to rescue and demographic/pre-operative clinical characteristics

Characteristic	All admissions (*N* = 374)	Survived (*N* = 286)	FTR (*N* = 88)	Relationship between characteristics and FTR (results from logistic regression)				
				Unadjusted	Adjusted^***^			
	n(column %)	n(row %)	n(row %)	*p*-value	OR	95% CI		*p*-value
Age (years)								
Median [P25, P75]	66 [54, 75]	64 51, 72]	73 [66, 80]	< 0.001	1.02	1.00 – 1.05		0.102
*Categorised age*: n(%)								
≤ 50	72 (19.3)	69 (95.8)	3 (4.2)	Ref	Ref			
51—60	61 (16.3)	54 (88.5)	7 (11.5)	0.127	2.30	0.41 – 12.83		0.343
61—70	109 (29.1)	80 (73.4)	29 (26.6)	0.001	4.33	0.81 – 23.14		0.086
71—80	87 (23.3)	59 (67.8)	28 (32.2)	< 0.001	3.14	0.54 – 18.26		0.202
> 80	45 (12.0)	24 (53.3)	21 (46.7)	< 0.001	4.21	0.65 – 27.18		0.131
*Gender: n (%)*								
Male	219 (58.6)	167 (76.3)	52 (23.7)	Ref	Ref			
Female	155 (41.4)	119 (76.8)	36 (23.2)	0.907	0.91	0.49 – 1.69	0.758	
BMI^*^								
Mean (SD)	27.8 (6.7)	28.1 (6.9)	26.4 (5.7)	0.046	0.96	0.91 – 1.01	0.116	
*Categorised BMI*: n(%)								
< 18.5 (underweight)	12 (3.4)	10 (83.3)	2 (16.7)	0.586	0.45	0.05 – 3.92	0.473	
18.5 – 24.9 (normal weight)	122 (34.4)	93 (76.2)	29 (23.8)	Ref	Ref			
25 – 29.9 (overweight)	100 (28.2)	78 (78.0)	22 (22.0)	0.756	0.99	0.48 – 2.06	0.981	
30 – 39.9 (obese)	102 (28.7)	85 (83.3)	17 (16.7)	0.191	0.60	0.26 – 1.36	0.218	
≥ 40 (morbidly obese)	19 (5.4)	16 (84.2)	3 (15.8)	0.444	0.86	0.22 – 3.34	0.830	
Smoking status ^**^: n(%)								
Never smoked	154 (41.2)	112 (72.7)	42 (27.3)	Ref	Ref			
Ex-smoker	148 (39.6)	114 (77.0)	34 (23.0)	0.390	0.75	0.39 – 1.45	0.393	
Current smoker	67 (17.9)	58 (86.6)	9 (13.4)	0.029	0.44	0.17 – 1.15	0.093	
*Cancer patient: n(%)*								
No	193 (51.6)	140 (72.5)	53 (27.5)	Ref	Ref			
Yes	181 (48.4)	146 (80.7)	35 (19.3)	0.067	0.37	0.19 – 0.69	0.002	
*Hypertension: n(%)*								
No	159 (42.5)	135 (84.9)	24 (15.1)	Ref	Ref			
Yes	215 (57.5)	151 (70.3)	64 (29.7)	0.001	1.66	0.82– 3.38	0.161	
*Pre-operative lymphocyte level (*× *10*^*3*^* cells/µL): n(%)*								
< 1.5	238 (63.8)	175 (73.5)	63 (26.5)	0.056	0.92	0.47 – 1.79	0.797	
≥ 1.5	135 (36.2)	111 (82.2)	24 (17.8)	Ref	Ref			
*Charlson comorbidity index*								
Median [P25, P75]	4 [2, 3]	4 [1, 5]	6 [4, 7]	< 0.001	1.30	1.12 – 1.50	< 0.001	
*Categorised CCI*								
Score < 3	119 (31.8)	111 (93.3)	8 (6.7)	Ref	Ref			
Score ≥ 3	255 (68.2)	175 (68.6)	80 (31.4)	< 0.001	3.68	1.15 – 11.74	0.028	
*Pre-operative albumin (g/L)*								
Mean (SD)	31 (6)	32 (6)	30 (7)	0.015	0.94	0.90 – 0.98	0.008	
*Categorised albumin*: n(%)								
< 18	8 (2.1)	2 (25.0)	6 (75.0)	< 0.001	8.46	1.06 – 67.56	0.044	
18 – 25	57 (15.2)	43 (75.4)	14 (24.6)	0.063	2.97	1.06 – 8.29	0.038	
26 – 30	104 (27.8)	76 (73.1)	28 (26.9)	0.016	3.19	1.29– 7.89	0.012	
31—34	91 (24.3)	69 (75.8)	22 (24.2)	0.046	2.25	0.86 – 5.91	0.099	
35 – 39 (normal)	76 (20.3)	67 (88.2)	9 (11.8)	Ref	Ref			
> 39	38 (10.2)	29 (76.3)	9 (23.7)	0.109	1.51	0.38 – 6.00	0.558	

Abbreviations: BMI—body mass index, SD—standard deviation, P25—25th percentile, P75—75th percentile, Ref—reference level for categorical variables

^*^BMI data missing at 19 patient surgeries

^**^Smoking status was unknown for 5 admissions

^***^The adjusted model included age, gender, body mass index, smoking status, cancer and hypertension diagnosis, pre-operative lymphocytes and albumin, ASA scores and Charlson Comorbidity Index

### Perioperative risk factors

Patients requiring ICU admission and who sustained intraoperative blood loss more than 500 mls had higher FTR rate. The FTR rate varied by combinations of surgery type and cancer diagnosis (interaction between surgery and cancer diagnosis (OR = 3.7; 95% CI 0.88–16; *p* = 0.075).

Most resections performed were for colorectal disease, followed by oesophagogastric, pancreatic and liver resections. The respective FTR for each group was 25%, 29%, 16% and 8.0% (Table 2).

**Table 2:** Summary of FTR and mortality by surgical speciality

	Total	CDC 3–5 (%)	FTR (%)	Mortality (%)
Colorectal	1599	17.57 (281)	24.91 (70)	4.38 (70)
Oesophagogastric	297	9.43 (28)	28.57 (8)	2.69 (8)
Pancreas	225	14.22 (32)	15.63 (5)	2.22 (5)
Liver	134	18.66 (25)	8.00 (2)	1.49 (2)
Trauma laparotomy	27	11.11 (3)	33.33 (1)	3.70 (1)

### Post-operative risk factors

The development of end-organ failure (Table 3), including the need for prolonged ventilation, inotropic support and/or dialysis/haemofiltration (Supplemental Table 1), significantly increased the mortality. There were 8 deaths due to medical comorbidities—four patients from stroke, two from pulmonary embolus and two from acute myocardial infarction.

**Table 3:** Relationship between initial surgery factors and failure to rescue (FTR)

Characteristic	All patients *N* = 374	Survived *N* = 286	FTR *N* = 88	Relationship between characteristics and FTR (results from logistic regression)				
				Unadjusted		Adjusted^**^		
	*n(column %)*	*n(row %)*	*n(row %)*	*p*-value	OR	95% CI	*p*-value	
ASA^*^								
1 or 2	132 (35.3)	124 (93.9)	8 (6.1)	Ref	Ref			
3	164 (43.9)	124 (75.6)	40 (24.4)	< 0.001	2.50	0.92 – 6.79	0.072	
4	47 (12.6)	19 (40.4)	28 (59.6)	< 0.001	11.90	3.29 – 42.95	< 0.001	
5	11 (2.9)	3 (27.3)	8 (72.7)	< 0.001	19.74	2.72 – 143.50	0.003	
Not stated	20 (5.3)	16 (80.0)	4 (20.0)	0.044	3.04	0.62 – 15.02	0.171	
*End of organ failure (only for ASA* ≥ *3)*								
No	168 (76.0)	127 (75.6)	41 (24.4)	Ref	Ref			
Yes	53 (24.0)	18 (34.0)	35 (66.0)	< 0.001	5.61	2.48 – 12.70	< 0.001	
*ICU admission*								
No	155 (41.4)	135 (87.1)	20 (12.9)	Ref	Ref			
Yes	219 (58.6)	151 (69.0)	68 (31.0)	< 0.001	2.50	1.13 – 5.53	0.024	
	Planned	131 (59.8)	100 (76.3)	31 (23.7)	Ref	Ref		
	Unplanned	88 (40.2)	51 (58.0)	37 (42.0)	0.004	2.19	0.97 – 4.90	0.058
	Length of stay: median [P25, P75]	4 [2, 7]	4 [2, 7]	4 [2, 7]	0.144	0.98	0.91 – 1.05	0.514
*Surgery type*								
Elective	255 (68.2)	220 (86.3)	35 (13.7)	Ref	Ref			
Emergency	119 (31.8)	66 (55.5)	53 (44.5)	< 0.001	3.35	1.68 – 6.70	0.001	
*Surgery type by cancer diagnosis*								
*No cancer*								
	Elective	101 (52.3)	92 (91.1)	9 (8.9)	Ref	Ref		
	Emergency	92 (47.7)	48 (52.2)	44 (47.8)	< 0.001	4.94	1.43 – 17.00	0.011
*Had cancer*								
	Elective	154 (85.1)	128 (83.1)	26 (16.9)	Ref	Ref		
	Emergency	27 (14.9)	18 (66.7)	9 (33.3)	0.053	1.89	0.68 – 5.27	0.223
*Elective surgery*								
	No cancer	101 (39.6)	92 (91.1)	9 (8.9)	Ref	Ref		
	Had cancer	154 (60.4)	128 (83.1)	26 (16.9)	0.075	1.45	0.57 – 3.68	0.431
*Emergency surgery*								
	No cancer	92 (77.3)	48 (52.2)	44 (47.8)	Ref	Ref		
	Had cancer	27 (22.7)	18 (66.7)	9 (33.3)	0.187	0.26	0.07 – 0.95	0.041
*Duration of surgery*								
	≤ 4 h	205 (54.8)	148 (72.2)	57 (27.8)	Ref	Ref		
	4–8 h	138 (36.9)	116 (84.1)	22 (15.9)	0.011	0.85	0.42 – 1.71	0.642
	> 8 h	31 (8.3)	22 (71.0)	9 (29.0)	0.887	3.63	1.12 – 11.73	0.031
*Intraoperative blood loss*								
No	319 (85.3)	249 (78.1)	70 (21.9)	Ref	Ref			
Yes	55 (14.7)	37 (67.3)	18 (32.7)	0.079	2.33	0.94 – 5.77	0.069	

Abbreviations: ASA—American Society of Anesthesiologists Classification, ICU—intensive care unit, P25–25th percentile, P75–75th percentile, Ref—reference level for categorical variables

^*^ASA scores not reported for 20 patients (16 survivors and 4 deceased patients)

^**^Adjusted for age, gender, body mass index, smoking status, cancer and hypertension diagnosis, pre-operative lymphocytes and albumin, ASA scores and Charlson Comorbidity Index

Of the 374 patients who suffered CDC 3–5 complications, 244 (59%) had at least one unplanned return to theatre (RTT) for their complication; the others were treated non-operatively. Patients with a higher BMI and those with a malignant diagnosis were more likely to require an RTT. (Supplemental Table 2).

## Discussion

This study is the first Australian study to examine pre-operative patient characteristics and their association with failure to rescue after surgery. A major strength of this study is that we captured all deaths within 30 days and in-hospital, and not just the 30-day mortality. Several pre-operative factors were associated with FTR, including comorbidities, cancer diagnosis and pre-operative albumin levels. Additionally, ASA score, need for ICU admission, surgery type and length were significantly associated with FTR in the event of complications. Patients admitted to ICU who suffered surgical complications requiring prolonged ventilation, inotropic support and/or dialysis were also more likely to die. RTT was performed for about two-thirds of patients suffering complications.

The ability of ASA score, comorbidities and serum albumin to predict failure to rescue suggests there is an opportunity, at least for higher risk patients undergoing planned (elective) surgery, to have their comorbidities optimised during the pre-operative period. For example, in the case of low serum albumin due to nutritional impairment, some patients may benefit from dietary advice and nutritional supplementation. Other cardiac, respiratory or metabolic comorbidities may also benefit from pre-operative optimisation [37]. Moderate risk patients, who do not necessarily need to be managed in ICU, may benefit from higher level care such as advanced recovery room care, identifying cardiovascular instability early, and proactively managing complications early to achieve better outcomes [25, 37].

We did not utilise the National Emergency Laparotomy Audit (NELA) Score, Portsmouth Physiological and Operative Severity Score (P-POSSUM) nor the American College of Surgeon National Surgical Quality Improvement Program (ACS-NSQIP), which have been shown to correlate with outcome for patients undergoing emergency laparotomy [38]. We acknowledge that NELA, P-POSSUM and ACS-NSQIP are sensitive enough to identify high-risk patients, but in the Australian context, they have been applied to emergency laparotomy. Our study included all patients undergoing major abdominal surgery with complications, not just emergencies or those who required a return to theatre. The Charlson Comorbidity Index is of proven predictive value as an accurate prognosticator of long-term mortality has been confirmed in many studies involving millions of patients [39]. A CCI is also calculable from Australian administrative data sets so could be used to report risk-adjusted outcomes and failure to rescue rates. We have instituted a further study specifically focused on emergency laparotomy and the ability of NELA and P-POSSUM to predict mortality. Unfortunately, our hospital does not have access to ACS-NSQIP unlike the Hunter Hospital and others in New South Wales.

The FTR rate observed in the current study was high compared to that from some large cohort international studies such as Ahmad et al. [40] who described an overall FTR of 2.8% (40), while Barmparas et al. [16] observed an FTR of 8.8% [41]. However, their studies also included complications with CDC scores 1 and 2. Our study only included Clavien–Dindo 3–5 patients, thus who require some intervention, and who have by definition an increased risk of RTT and mortality. Peacock et al. [42] in their study of FTR after emergency laparotomy for large bowel perforation reported a FTR-surgical rate of 16.8% (*n* = 172) [42], while ours was similar at 16.7% (*n* = 47) for all colorectal emergencies. The significant association between comorbidities and FTR is similar to what was observed by Pandit et al. [43] in a retrospective study of 49, 000 patients undergoing colectomies. They noted that 20% of patients had a CCI of $\ge$ 3 and further concluded CCI to be an independent risk factor for FTR [43]. Gleeson et al. [44] also found, similar to our study, that hypoalbuminemia is a significant predictor of FTR.

One limitation of this study is that we excluded CDC 1 and 2 complications, therefore under recorded medical complications (i.e., infections, acute kidney injury) and their management. The ability to recognise deterioration and appropriately escalate care has a major impact on the ability to rescue any patient suffering complications from surgery, whether surgical or medical. We also acknowledge the cases that we excluded such as laparoscopic appendicectomy, cholecystectomy and ventral hernia without laparotomy also suffer complications. However, this exclusion was deliberate to study a more severe cohort of patients, the ones most at risk of dying.

Factors such as low nurse-to-patient ratio [45, 46], high burnout rate [46] and lower patient socioeconomic status [47] have been previously reported as predictors of FTR in American studies. Given this was a pre-COVID-19 pandemic series, these factors are likely to have been stable, though were not included for our cohort, given the patients were recruited from one surgical programme. A further study looking into health services comparison and socioeconomic profiles in the Australian and New Zealand context would be useful. Further, a higher mortality has been observed in lower volume hospitals and significantly [48–50] with hospital volume contributing more significantly to FTR compared to post-operative complication rates [51]. Ghaferi et al. found that low-volume hospitals had markedly higher FTR compared with higher volume hospitals (30.3% Vs 13.1%) [51]. Within the Australian and New Zealand context, and for the operations included, registry and/or health department data show our hospital is regarded as high volume for colorectal surgery and emergency laparotomy, and medium volume for oesophagogastric, hepatic and pancreaticoduodenal surgery.

This study indicates that pre-, peri- and post-operative factors are associated with the likelihood of FTR in the event of complications. This understanding can improve informed shared decision-making prior to major abdominal surgery and the subsequent management of any complications. Patients at high risk of FTR and their families will benefit from a frank discussion regarding the appropriateness of operative intervention, understanding their goals of care, as well as the risks for morbidity and/or mortality.

## Supplementary Information

Below is the link to the electronic supplementary material.

Supplementary file1 (DOCX 19 kb)
