# Supplementary material for: Failure to Rescue in Major Abdominal Surgery: A Regional Australian Experience
Source: World J Surg. 2023 May 24;47(9):2145–53. doi: 10.1007/s00268-023-07061-x (PMC10208200; doi:10.1007/s00268-023-07061-x)
Supplement: Supplementary file 1 — Supplementary file1 (DOCX 19 kb) [file 268_2023_7061_MOESM1_ESM.docx]

**Supplementary Tables**

***Supplemental Table 1: Relationship between complications following initial surgery and FTR***

| **Characteristic** | **All patients**  **N=374** | **Survived**  **N=286** | **FTR**  **N=88** | **Comparison between groups** |
| --- | --- | --- | --- | --- |
|  | *n(column %)* | *n(row %)* | *n(row %)* |  |
| **ASA (post-surgical)** |  |  |  |  |
| 1 or 2 | 46 (12.3) | 44 (95.7) | 2 (4.3) | <0.001 |
| 3 | 94 (25.1) | 86 (91.5) | 8 (8.5) |  |
| 4 | 63 (16.8) | 43 (68.3) | 20 (31.7) |  |
| 5 | 7 (1.9) | 2 (28.6) | 5 (71.4) |  |
| Not reported | 164 (43.9) | 111 (67.7) | 53 (32.3) |  |
| **Type of RTT^*^** |  |  |  |  |
| Emergency | 177 (47.3) | 148 (83.6) | 29 (16.4) | <0.001 |
| Planned | 59 (15.8) | 49 (83.1) | 10 (17.0) |  |
| Radiological intervention | 31 (8.3) | 30 (96.8) | 1 (3.2) |  |
| Medical treatment only including IV antibiotics and transfusion | 73 (19.5) | 57 (78.1) | 16 (21.9) |  |
| Palliation | 31 (8.3) | 2 (6.5) | 29 (93.6) |  |
| **Type of intervention** |  |  |  |  |
| Laparoscopic | 41 (11.0) | 33 (80.5) | 8 (19.5) | 0.201 |
| Open | 166 (44.4) | 135 (81.3) | 31 (18.7) |  |
| Drain/Debride/VAC | 35 (9.4) | 33 (94.3) | 2 (5.7) |  |
| Not stated | 132 (35.3) | 85 (64.4) | 47 (35.6) |  |
| **Dialysis** |  |  |  |  |
| Not stated | 78 (20.9) | 55 (70.5) | 23 (29.5) |  |
| No | 277 (74.1) | 224 (80.9) | 53 (19.1) | <0.001 |
| Yes | 19 (5.1) | 7 (36.8) | 12 (63.2) |  |
| **ICU admission** |  |  |  |  |
| Not stated | 13 (3.5) | 8 (61.5) | 5 (38.5) |  |
| No | 212 (56.7) | 175 (82.6) | 37 (17.5) | 0.003 |
| Yes | 149 (39.8) | 103 (69.1) | 46 (30.9) |  |
| Planned | 90 (60.4) | 58 (64.4) | 32 (35.6) | 0.130 |
| Unplanned | 59 (39.6) | 45 (76.3) | 14 (23.7) |  |
| **Prolonged ventilation ^**^** |  |  |  |  |
| No | 51 (34.2) | 46 (90.2) | 5 (9.8) | <0.001 |
| Yes | 97 (65.1) | 57 (58.8) | 40 (41.2) |  |
| Time under ventilation |  |  |  |  |
| <24hours | 22 (22.7) | 15 (68.2) | 7 (31.8) | 0.422 |
| 1-3 days | 29 (29.9) | 18 (62.1) | 11 (37.9) |  |
| > 3 days | 46 (47.4) | 24 (52.2) | 22 (47.8) |  |
| **Inotropic support ^**^** |  |  |  |  |
| No | 62 (41.6) | 52 (83.9) | 10 (16.1) | 0.002 |
| Yes | 86 (57.7) | 51 (59.3) | 35 (40.7) |  |
| Time under inotropic support |  |  |  |  |
| <24hours | 16 (18.6) | 10 (62.5) | 6 (37.5) | 0.319 |
| 1-3 days | 34 (39.5) | 23 (67.7) | 11 (32.4) |  |
| > 3 days | 36 (41.9) | 18 (50.0) | 18 (50.0) |  |

Abbreviations: ASA- American Society of Anesthesiologists Classification, ICU – intensive care unit, RTT – return-to-theatre, FTR – failure to rescue

^*^ Data missing for three patients.

^**^ Only reported for those admitted into ICU. Data missing for one patient.

***Supplemental Table 2: Relationship between return-to-theatre (RTT) and patient characteristics at the time of initial surgery (exclude this table from supplementary table)***

| **Characteristic** | **No RTT**  **N=130** | **RTT**  **N=244** | **Relationship between characteristics and RTT (results from Logistic regression)** | | | |
| --- | --- | --- | --- | --- | --- | --- |
|  |  |  | **Unadjusted** | **Adjusted^**^** | | |
|  |  |  | **p-value** | **OR** | **95% CI** | **p-value** |
| **Age** (years): Median[P25, P75] | 67 [53, 77] | 65 [54, 74] | 0.360 | 1.00 | 0.98 – 1.02 | 0.940 |
| **Gender:** n(%) |  |  |  |  |  |  |
| Male | 76 (34.7) | 143 (65.3) | Ref | Ref |  |  |
| Female | 54 (34.8) | 101 (65.2) | 0.978 | 0.84 | 0.52 – 1.35 | 0.478 |
| **BMI** (kg/m^2^) |  |  |  |  |  |  |
| Mean (SD) | 26.7 (6.2) | 28.3 (6.8) | 0.035 | 1.04 | 1.00 – 1.08 | 0.027 |
| *Categorised BMI*: n(%) |  |  |  |  |  |  |
| < 18.5 (underweight) | 6 (50.0) | 6 (50.0) | 0.514 | 0.70 | 0.24 – 2.04 | 0.515 |
| 18.5 – 24.9 (normal weight) | 50 (41.0) | 72 (59.0) | Ref | Ref |  |  |
| 25 – 29.9 (overweight) | 30 (30.0) | 70 (70.0) | 0.095 | 1.55 | 0.86 – 2.79 | 0.144 |
| 30 – 39.9 (obese) | 29 (28.4) | 73 (71.6) | 0.054 | 1.77 | 0.97 – 3.22 | 0.063 |
| ≥40 (morbidly obese) | 6 (31.6) | 13 (68.4) | 0.439 | 1.64 | 0.51 – 5.25 | 0.408 |
| **Smoking status:** n(%) |  |  |  |  |  |  |
| Never smoked | 54 (35.1) | 100 (64.9) | Ref | Ref |  |  |
| Ex-smoker | 48 (32.4) | 100 (67.6) | 0.635 | 1.14 | 0.66 – 1.97 | 0.641 |
| Current smoker | 25 (37.3) | 42 (62.7) | 0.747 | 1.11 | 0.57 – 2.15 | 0.764 |
| **Charlson comorbidity index** |  |  |  |  |  |  |
| Median[P25, P75] | 4 [2, 7] | 4 [2, 6] | 0.387 | 0.99 | 0.86 – 1.15 | 0.940 |
| *Categorised CCI*: n(%) |  |  |  |  |  |  |
| Score < 3 | 43 (36.1) | 76 (63.9) | Ref | Ref |  |  |
| Score ≥ 3 | 87 (34.1) | 168 (65.9) | 0.707 | 1.78 | 0.87 – 3.64 | 0.116 |
| **Pre-operative albumin** |  |  |  |  |  |  |
| Mean(SD) | 31 (6) | 32 (6) | 0.540 | 1.00 | 0.96 – 1.03 | 0.863 |
| *Categorised albumin*: n(%) |  |  |  |  |  |  |
| < 35 | 92 (35.4) | 168 (64.6) | 0.849 | 1.23 | 0.69 – 2.20 | 0.488 |
| 35 – 39 | 26 (34.2) | 50 (65.8) | Ref | Ref |  |  |
| >39 | 12 (31.6) | 26 (68.4) | 0.779 | 1.61 | 0.65 – 3.98 | 0.306 |
| **Cancer patient by surgery type** |  |  |  |  |  |  |
| Elective |  |  |  |  |  |  |
| No | 39 (38.6) | 62 (61.4) | Ref | ref |  |  |
| Yes | 46 (29.9) | 108 (70.1) | 0.155 | 1.94 | 1.01 – 3.71 | 0.045 |
| Emergency |  |  |  |  |  |  |
| No | 36 (39.1) | 56 (60.9) | Ref | Ref |  |  |
| Yes | 9 (33.3) | 18 (66.7) | 0.586 | 1.09 | 0.36 – 3.31 | 0.882 |
| No cancer |  |  |  |  |  |  |
| Emergency | 36 (39.1) | 56 (60.9) | Ref | Ref |  |  |
| Elective | 39 (38.6) | 62 (61.4) | 0.942 | 0.59 | 0.26 – 1.34 | 0.206 |
| Has cancer |  |  |  |  |  |  |
| Emergency | 9 (33.3) | 18 (66.7) | Ref | Ref |  |  |
| Elective | 46 (29.9) | 108 (70.1) | 0.717 | 1.23 | 0.50 – 3.02 | 0.658 |
| **Hypertension** |  |  |  |  |  |  |
| No | 52 (32.7) | 107 (67.3) | Ref | Ref |  |  |
| Yes | 78 (36.3) | 137 (63.7) | 0.475 | 0.84 | 0.49 – 1.44 | 0.528 |
| **Pre-operative lymphocyte level** |  |  |  |  |  |  |
| < 1.5 | 91 (38.2) | 147 (61.8) | 0.046 | 1.55 | 0.94 – 2.55 | 0.088 |
| ≥ 1.5 | 38 (28.2) | 97 (71.9) | Ref | Ref |  |  |
| **ASA** |  |  |  |  |  |  |
| 1 or 2 | 41 (31.1) | 91 (68.9) | Ref | Ref |  |  |
| 3 | 55 (33.5) | 109 (66.5) | 0.655 | 0.98 | 0.54 – 1.77 | 0.940 |
| 4 | 23 (48.9) | 24 (51.1) | 0.030 | 0.47 | 0.20 – 1.09 | 0.078 |
| 5 | 5 (45.5) | 6 (54.5) | 0.333 | 0.73 | 0.17 – 3.22 | 0.678 |
| Not stated | 6 (30.0) | 14 (70.0) | 0.924 | 1.35 | 0.46 – 3.95 | 0.583 |

Abbreviations: ASA- American Society of Anaesthesiologists Classification, ICU – intensive care unit, BMI – body mass index, SD – standard deviation, P25 – 25^th^ percentile, P75 – 75^th^ percentile
